# CDK4/6 Inhibitors-Induced Macrocytosis Is Not Associated with Hemolysis and Does Not Impact Hemoglobin Homeostasis

**DOI:** 10.3390/cancers17091567

**Published:** 2025-05-05

**Authors:** Tiago Barroso, Leila Costa, Lisa Gonçalves, Vanessa Patel, João Araújo, Inês Pinho, Carolina Monteiro, Miguel Esperança-Martins, Catarina Abreu, Rita Teixeira de Sousa, Helena Pais, Gonçalo Nogueira-Costa, Sofia Torres, Leonor Abreu Ribeiro, Luís Marques da Costa

**Affiliations:** 1 Medical Oncology Department, Unidade Local de Saúde Santa Maria, 1649-03 Lisbon, Portugal; lisa.goncalves@ulssm.min-saude.pt (L.G.); vanessa.patel@ulssm.min-saude.pt (V.P.); joao.e.araujo@ulssm.min-saude.pt (J.A.); ines.pinho@ulssm.min-saude.pt (I.P.); carolina.monteiro@ulssm.min-saude.pt (C.M.); miguelmemartins@campus.ul.pt (M.E.-M.); catarina.abreu@ulssm.min-saude.pt (C.A.); ana.r.sousa@ulssm.min-saude.pt (R.T.d.S.); helena.pais@ulssm.min-saude.pt (H.P.); goncalo.costa@ulssm.min-saude.pt (G.N.-C.); sofia.torres@ulssm.min-saude.pt (S.T.); leonor.ribeiro@ulssm.min-saude.pt (L.A.R.); luis.costa@ulssm.min-saude.pt (L.M.d.C.); 2 Pharmacy Department, Unidade Local de Saúde Santa Maria, 1649-03 Lisbon, Portugal; leila.costa@ulssm.min-saude.pt; 3 Luís Costa Lab, Instituto de Medicina Molecular JLA, 1649-028 Lisbon, Portugal

**Keywords:** breast cancer, endocrine therapy, CDK4/6 inhibitor, red blood cell, hematological toxicity

## Abstract

Background: CDK 4/6 inhibitors (CDK4/6is) are the first-line treatment for metastatic luminal-like breast cancer (BC). These drugs induce macrocytosis without anemia in most patients. The mechanism for the red blood cell (RBC) changes is unknown. In vitro and animal studies show that RBCs from CDK6-knockout mice have increased membrane fragility, but the clinical impact of CDK4/6is on human RBC lifespan is not known. We sought to determine the impact of CDK4/6is on RBC lifespan and detect changes in the regulation of hemoglobin production. Using the mean corpuscular volume (MCV) measurements at several time points, we can study the evolution of MCV, mean corpuscular hemoglobin concentration (MCHC), and RBC count over time. From this, one can estimate the RBC lifespan under CDK4/6is. Methods: We performed a unicentric retrospective study. Based on published models of RBC population dynamics, we have coded a biologically inspired model which allowed us to extract values for biological parameters, including the RBC lifespan. Results: A total of 122 patients were identified, and 1959 laboratory measurements were analyzed. After the pre-treatment RBCs were replaced, the mean MCV increased by 12.6 femtoliter (fL) (95% Bayesian credible interval [CdI] 13–14), the MCHC increased slightly by 0.69 g/dL (95% CdI 0.42–0.96), and the RBC count decreased by 0.77 × 10^9^/L (95% CdI 0.42 × 10^9^/L–0.96 × 10^9^/L). The net result was a 0.64 g/dL (95% CdI 0.48–0.80) rise in hemoglobin. The mean total RBC lifetime was 118 days (95% CdI 114–122), similar to the value measured in healthy persons. Discussion and Conclusions: These findings suggest that, despite changes in RBC volume, CDK4/6is do not predispose patients to RBC destruction and do not impair regulation of hemoglobin homeostasis. We show that CDK4/6is do not decrease the RBC lifespan in pre-treatment erythrocytes. Unfortunately, this method cannot determine the lifespan of post-treatment RBCs, but further research could help answer this question.

## Simple Summary

CDK4/6 inhibitors are drugs used to treat breast cancer. These drugs tend to increase the size of red blood cells with prolonged use, and the significance of these changes is unknown. Despite data from animal experiments, it was not known whether such size changes could cause red blood cells to be destroyed in humans. In this study, we use data from patient’s blood tests to show that CDK4/6 inhibitors are not associated with premature red blood cell destruction in human patients and we highlight directions for future research.

## 1. Introduction

Oral selective cyclin-dependent kinase 4/6 inhibitors (CDK4/6is) are currently part of the first-line treatment for metastatic luminal-like breast cancer [1,2]. The three CDK4/6is approved for use in clinical practice for first-line treatment of advanced estrogen receptor-positive, HER2-negative breast cancer together with endocrine therapy (palbociclib, ribociclib, and abemaciclib) are associated with an increase in progression-free survival [3,4,5]. However, only the MONALEESA-2 trial has shown an overall survival advantage of ribociclib plus endocrine therapy compared to endocrine therapy alone [5]. Data from the PALOMA and Monarch trials have failed to show an overall survival advantage of either palbociclib or abemaciclib [3,4].

More recent trials, such as the MonarchE [6] and the NATALEE [7] trials, have tested the effectiveness of ribociclib and abemaciclib in the adjuvant setting. Both trials have shown an increase in disease-free survival, but data are still too immature to evaluate the impact on overall survival. Most of the above-mentioned trials have studied the corresponding CDK4/6i in combination with aromatase inhibitors. Clinical data have thus shown an in vivo additive effect between the aromatase inhibitor and CDK4/6is. On the other hand, in vitro data show that palbociclib is also synergistic with the selective estrogen receptor modulator tamoxifen in inhibiting the growth of an estrogen-positive breast cancer cell line [8]. Interestingly, despite the additive therapeutic effects, in the adjuvant setting, a recent meta-analysis has shown that concomitant CDK4/6i use might be protective of the musculo-skeletal side effects of aromatase inhibitors [9].

Amongst the most common side effects of CDK4/6is are hematological side effects. Palbociclib and ribociclib are associated with neutropenia and, to a smaller degree, anemia and thrombocytopenia. The severity of neutropenia is highly variable among patients. Neutropenia is managed with dose reductions. Abemaciclib, possibly because of different affinities to CDK4, CDK6, and CDK2, has fewer hematological side effects and a higher frequency and severity of gastrointestinal side effects such as nausea and diarrhea, which can usually be managed with symptomatic therapy [10].

Anemia and thrombocytopenia have not been as well-studied as neutropenia in patients taking ribociclib, palbociclib, and abemaciclib. These side effects are much rarer than neutropenia, and it is not clear whether clinically significant anemia is a direct consequence of the mechanism of action of these drugs or whether it is an idiosyncratic reaction in some patients. Although anemia is rare, ribociclib, abemaciclib, and palbociclib tend to induce red blood cell macrocytosis without anemia in most patients [11]. The mechanism for the red blood cell (RBC) changes under CDK4/6 inhibition is currently unknown. In particular, it is not known if these changes are associated with myeloid lineage dysplasia in most patients, as blood smears are only typically performed when overt dysplastic anemia is present. In vitro studies show that CDK6-knockout mouse RBCs have increased membrane fragility due to CDK6-dependent cytoskeletal changes [12,13]. Aside from that, CDKi 4/6 inhibitors tend to cause significant anemia in mice. These pre-clinical data suggest that inhibition of CDK6 could lead to increased hemolysis in vivo, thus decreasing red blood cell lifespan in human patients [13]. The fact that anemia is rare in these patients cannot be used to rule out this hypothesis, as a decreased RBC lifespan can be compensated by increased RBC production. Additionally, some recent pre-clinical work has not found increased hematological toxicity in mice [14].

An understanding of how CDK4/6i use impacts red blood cell parameters, such as mean corpuscular volume (MCV), mean corpuscular hemoglobin concentration (MCHC), red blood cell numbers, and red blood cell lifespan can lead to a better understanding of the more subtle ways in which these drugs impact important homeostatic functions in the blood marrow and peripheral blood. Of these, the most clinically relevant parameter is the RBC lifespan. A lower RBC lifespan (for example, due to decreased cytoskeletal stability as mentioned above) can result in a state of compensated hemolysis or hemolytic anemia. As the use of CDK4/6is expands, particularly in the adjuvant setting [6,7], where patients do not have overt disease and no symptoms attributable to the disease, elucidating these more subtle forms of toxicity becomes even more relevant.

Our main goal was to determine whether the clinically used CDK4/6is (abemaciclib, palbociclib, and ribociclib) are associated with increased RBC destruction (or, equivalently, decreased RBC lifespan) in human subjects treated with the doses used in clinical practice.

## 2. Materials and Methods

### 2.1. Data Collection and Inclusion Criteria

We performed a retrospective study of breast cancer patients treated at our center with ribociclib, palbociclib, or abemaciclib between 2017 and 2022 for metastatic disease, for whom the starting date of the treatment was known and for whom at least five complete blood counts were available. We excluded patients who had received the CDK4/6is as part of a clinical trial. We also excluded patients who had high MCV at baseline (MCV > 98 femtoliter [fL]) due to the fact that such baseline values could indicate concurrent conditions capable of impacting RBC homeostasis. Potentially eligible patients were identified from pharmacy records. Laboratory measurements were extracted from the patients’ electronic medical records. We analyzed laboratory measurements from 4 weeks before the first dose of CDK4/6is until drug discontinuation or last follow-up date while still on the drug. Informed consent was waived by the Ethics committee given the retrospective nature of data collection.

### 2.2. Red Blood Cell Lifespan Estimation

Red blood cell lifespan can be measured directly with invasive methods for RBC labeling. The most common techniques involve labeling RBCs with radioactive cobalt or (non-radioactive) biotin [15]. These methods are cumbersome, unavailable in most clinical settings, and are ethically difficult in investigation with human subjects who are not expected to benefit directly from the test result. Less invasive methods relying on heme-bound carbon monoxide are also available, but they have not yet been properly validated in large populations and are not easily accessible in the clinic [16].

To estimate the RBC lifespan in clinical practice, one must resort to indirect methods. According to the current understanding of erythropoiesis, RBC volume appears to be approximately fixed from the time of final RBC maturation to the time of RBC death. From this, one can assume that the increase in RBC volume in patients under CDK4/6is inhibitors is caused by larger new RBCs created under the effect those drugs have on erythropoiesis and not as consequence of the already mature RBCs, as RBC volume tends to decrease and not increase during their lifetime [17]. Thus, according to our assumptions, RBCs created before the patient has started the CDK4/6i will be small (low MCV) and RBCs created after the patient has started the CDK4/6i will be large (high MCV).

**Figure 1:**
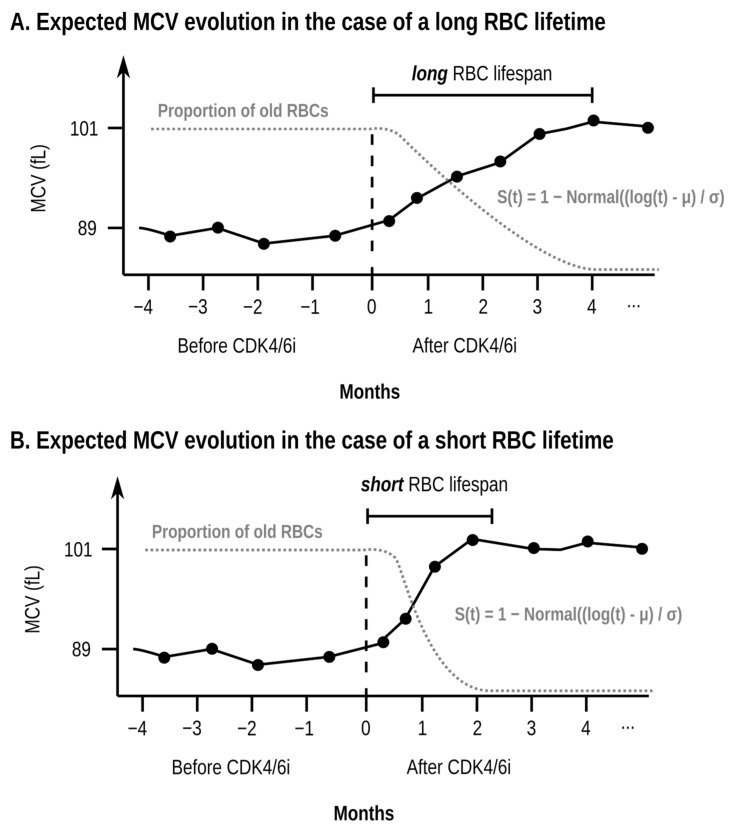
Illustrative schematic example (simulated data, not data taken from any specific patient) of the evolution of the mean corpuscular volume (MCV) over time for a single patient (black continuous line) and the proportion of old red blood cells (RBCs) (grey dotted line). The two subplots (**A**,**B**) show the expected effect of a longer (subplot (**A**)) and shorter (subplot (**B**)) RBC lifetime in the evolution of the MCV and the proportion of old RBCs. In the case of a long RBC lifetime (subplot (**A**)), the patient will take a long time to replace the pre-treatment RBCs (RBCs with a low MCV) with the new RBCs (RBCs with a high MCV). As a result, the proportion of old RBCs will decrease slowly and the MCV will grow slowly. In contrast, if the RBC lifetime is short (subplot (**B**)), the proportion of old RBCs will decrease rapidly, and the MCV will grow quickly. The horizontal axis shows the time in months using as a reference the time at which the patient starts taking the CDK4/6 inhibitor (CDK4/6i). The vertical axis shows both the MCV (in femtoliters, fL) and the proportion of old RBCs. The red blood cell (RBC) lifespan of the pre-treatment RBCs is the time which takes to replace 95% of the previous population of RBCs to be replaced by new RBCs created under the effect of treatment. The increase in MCV is proportional to the rate of replacement for the pre-treatment RBCs, which can be modeled using well-established equations, namely the S(t) function shown in the plot. Real patient data are noisy, and determining the RBC lifespan is hard at the single-patient level because of the high variability of MCV measurements. As such, in our model, the RBC lifespan is estimated as a single value for the whole population of patients.

The patient’s MCV will be a weighted average between the MCV of the small RBCs and the MCV of the large RBCs, according to the proportions of these populations. As time passes, the old (small) RBCs will be replaced by the new (large) RBCs according to population dynamics governed by equations that are currently well established [15]. Together with the knowledge of those equations, one can use the increase in MCV over time to estimate the RBC lifespan of RBCs created before the patient has started taking CDK4/6is. Intuitively, if the RBC lifetime is low, old (small) RBCs will be replaced very rapidly, and the MCV will increase will be faster. In contrast, if the RBC lifetime is high, the old RBCs will be replaced slowly and the rise in MCV will be slower as a result. Figure 1 shows in a graphical way how the RBC lifetime influences MCV growth. The equations in [15] allow us to be precise in the way these quantities relate to each other and as a result estimate the RBC lifetime (which is unknown) from the increase in MCV (which can be measured from the laboratory values). Because RBCs created under the effect of the CDK4/6is will be the same size, they will be indistinguishable for our purposes, and we will not be able to estimate their lifetime.

If the estimated RBC lifespan of these patients is lower than the normal 118 days from healthy subjects [15,18], this means that CDK4/6is have a detrimental effect on RBC lifespan. However, if the estimated value RBC lifespan for these patients is similar to the measured RBC lifespan for healthy subjects, one can conclude that despite the pre-clinical data shown above, CDK4/6is have no impact on the lifespan of pre-existing RBCs in humans at the doses used in clinical practice. However, our method does have the limitation that we cannot estimate the lifespan of RBCs created under the effect of the CDK4/6is. A different approach is required to estimate the approach of post-treatment RBCs, as we explain below.

Because patients treated with palbociclib, ribociclib, and abemaciclib take these drugs for a period of several months and have regular complete blood counts (bi-weekly for the first two months and then monthly afterward), one can use the laboratory data for these patients to fit a Bayesian model to the repeated MCV, MCHC, and RBC counts over time, and extract the relevant parameters from the model.

### 2.3. Bayesian Model Specification

The rationale for using a Bayesian model instead of the more common frequentist approach is twofold. First, using Bayesian inference allows for a simpler interpretation of the estimated parameters (credibility intervals) when compared to the frequent equivalent (confidence intervals) [19]. Second, the complex nature of the model, with hierarchical parameters, lends itself better to Hamiltonian Monte Carlo methods, which are better supported by Bayesian inference software such as Stan 2.31 [20]. A Bayesian approach also allows for using known information (such as the measured parameters for the RBC lifetime survival function) in the prior distribution for the relevant parameters. Based on data published on RBC population dynamics, we have coded a biologically inspired hierarchical Bayesian model in the Stan programming language.

To simplify the notation, we will refer to RBCs created before the patient started treatment with CDK4/6is as *old* RBCs and the RBCs created after as *new* RBCs. Under this naming convention, old RBCs will be small and new RBCs will be large. Based on the known equations, we will assume that the residual survival function *S* for old RBCs follows a LogNormal model. In this case, the function S is given by [15]:

S(*t*, *μ*_survival_, *σ*_survival_) = 1 − Normal((log(*t*) + *μ*_survival_)/*σ*_survival_)

where Normal(*x*) is the probability distribution function of the standard normal distribution.

Time t represents the number of days since the patient has started treatment. For the 1st day of treatment we will have *t* = 0, for the time period before treatment we will have *t* < 0, and for the time period after treatment we will have *t* > 0.

One can model the proportion of old RBCs (*w_old_* at time *t*) with the following equations:

*w*_old_(*t*) = 1.0 if *t* ≤ 0 (all RBCs are old before treatment starts)

*w*_old_(*t*) = *S*(*t*, *μ*_survival_, *σ*_survival_) if *t* > 0

Once the proportion of old RBCs has been defined as *w*_old_(*t*), the proportion of new RBCs at time *t* is given by 1 − *w*_old_(*t*). Figure 1 shows a hypothetical evolution of the MCV of a single patient as and how it relates to the fraction of surviving red blood cells.

We modeled the value at each time point *t* for each of the remaining parameters, namely, RBC number (*N*_RBC_) and mean corpuscular hemoglobin concentration (MCHC), mean corpuscular volume (MCV) as student T distributions (StudentT) with a known mean and unknown degrees of freedom (*υ*), and standard distribution (*σ*). The Student T distribution is better than the more commonly used Normal distribution at modeling noisy data such as the laboratory values from our patients. In particular, we have found that the Normal distribution fails to capture the high intra-patient variability of the laboratory measurements, which often show unexpectedly high or low values with higher frequency than what would be expected if the values were modeled with the Normal distribution. Simple algebra shows that the mean value for *N*_i_(t), MCHC_i_(t), and MCV_i_(t) is given by a weighted arithmetic average between the values for the old and for the new RBCs. We can formalize this as follows:

*N*_i_(t) ~ StudentT(*ζ*_N_, *w*_old_(*t*) N_RBC, i, old_ + (1 − *w*_old_(*t*)) N_RBC, i, new_, *σ*_N_)

MCHC_i_(t) ~ StudentT(*ζ*_MCHC_, *w*_old_(*t*) MCHC_i, old_ + (1 − *w*_old_(*t*)) MCHC_i, new_, *σ*_MCHC_)

MCV_i_(t) ~ StudentT(*ζ*_MCV_, *w*_old_(*t*) MCV_i, old_ + (1 − *w*_old_(*t*)) MCV_i, new_, *σ*_MCV_)

We model the RBC indices for each individual patient as being sampled from a normal distribution with unknown mean (*μ*) and standard deviation (*σ*). The patient-level parameters for the *i*-th patient are given by:

*N*_i, old_ ~ Normal(*μ*_N, old_, *σ*_N, old_)

*N*_i, new_ ~ Normal(*μ*_N, new_, *σ*_N, new_)

MCHC_i, old_ ~ Normal(*μ*_MCHC, old_, *σ*_MCHC, old_)

MCHC_i, new_ ~ Normal(*μ*_MCHC, new_, *σ*_MCHC, new_)

MCV_i, old_ ~ Normal(*μ*_MCV, old_, *σ*_MCV, old_)

MCV_i, new_ ~ Normal(*μ*_MCV, new_, *σ*_MCV, new_)

The main parameter of interest, the RBC lifespan, can be estimated from the *μ*_survival_ and *σ*_survival_ values according to the reference equations for the *S* function. Assuming a LogNormal distribution for RBC survival, the RBC lifespan is given by exp(*μ*_survival_ + *σ*_survival_^2^/2)), where exp(*x*) is the exponential function *e^x^*.

At each point in time, the proportion of surviving RBCs was modeled as a weighted average of the MCV of the pre-treatment RBCs and the post-treatment RBCs. The model was fit to all available complete blood count measurements for each patient, both before and after the patient had started taking the CDK4/6i. The use of this model-based approach allows us to incorporate laboratory data gathered at any point in time, avoiding complexities associated with discretizing time intervals. We fit the model to the data and extracted 95% Bayesian credible intervals (CdIs) for the model parameters, including the time delay. Note that the *before* and *after* parameter values are not measured at a specific cutoff in time but instead are evaluated from the (continuous) model equations and from the model fit to the whole available data.

Data cleaning and data analysis were performed in the Python programming language with the Numpy 1.21 [21], Matplotlib 3.5 [22], ArViz 0.20 [23], and CmdStanPy 1.1 [24]. The full code is available upon request.

### 2.4. Model Fit and Model Diagnostics

We have fit the model using Stan using the default settings of four parallel Hamiltonian Monte Carlo chains, with a total number of 4000 draws per chain, where the first 2000 draws were considered as warm-up draws and discarded. No divergences were found during sampling.

We report the posterior probabilities for all population-level parameters (including the full RBC lifetime) in the form of medians and 95% Bayesian credible intervals (CdIs). Besides reporting the medians and the 95% CdIs, we plot the posterior distribution for all four chains and present the rank plot for each parameter, to prove there was adequate mixing between chains (with the large number of draws, the trace plots are not as informative as the rank plots).

To assess goodness of fit, we plotted posterior predictive plots (PPCs) and the leave-one-out (LOO) plots for the RBC number, MCVl, and MCHC. To look for outliers we evaluated the $\hat{k}$ statistic for each blood draw. As prescribed by Vehtari et al. [25], we considered data points for which $\hat{k}$ > 1 as severe outliers.

## 3. Results

We identified a total of 122 patients. All patients were female. The median age was 59 (interquartile-range 49–70). The median duration of continuous CDK4/6 use was 7.2 months (interquartile range 3.4–13.3 months). Of these patients, 16 (13.1%) were treated with abemaciclib, 65 (53.3%) were treated with palbociclib, and 41 (33.6%) were treated with ribociclib (see Table 1 and the respective label for further details). A total of 1959 complete blood counts were included, a mean of 16 blood test results per patient.

**Figure 2:**
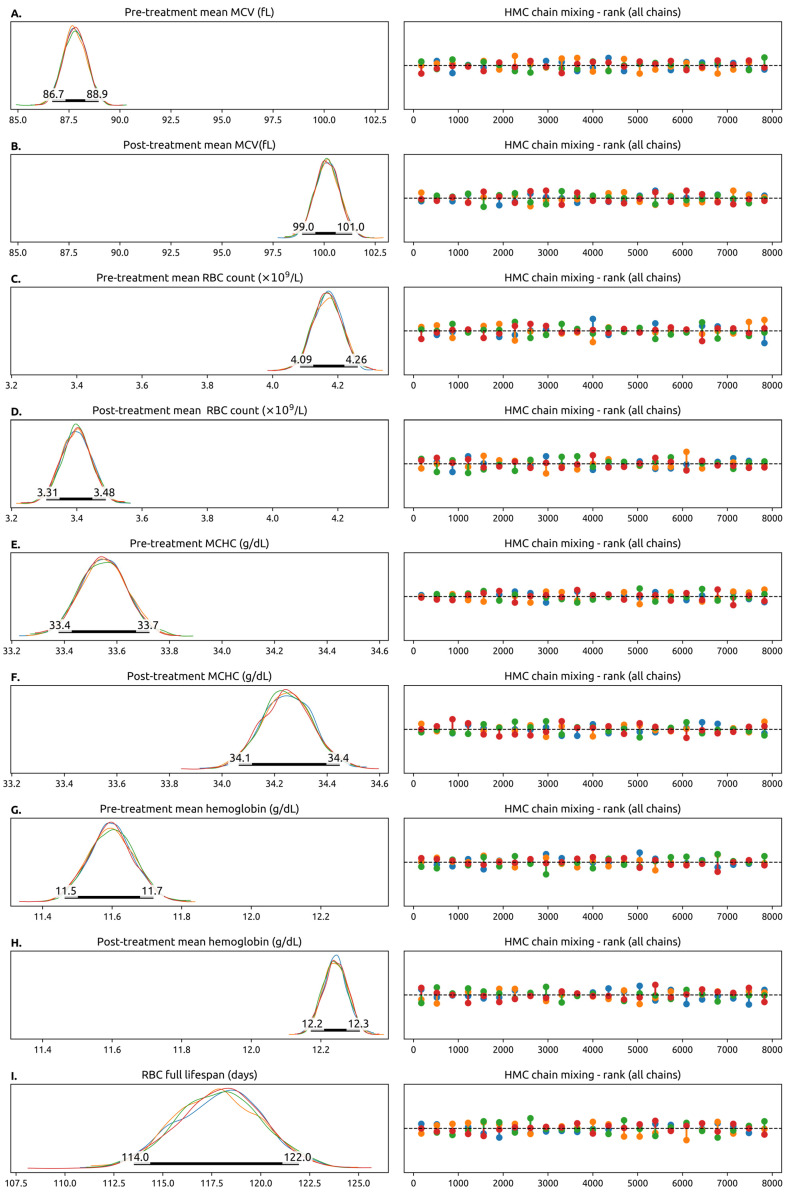
Trace plots of selected model parameters (some model parameters are not represented here). The left column contains the posterior probability distributions for the parameters estimated from the 4 parallel Hamiltonian Monte Carlo chains, each chain in a different color. In the Hamiltonian Monte Carlo simulation, each chain is simulated independently using different starting values for the parameters. If the model is well-specified and matches the experimental data, the chains should converge into the same distribution. The number of simulated chains is somewhat arbitrary, and we decided to simulate 4 chains as that is considered a sensible default in a number of software packages. The fact that in general the chains overlap means that they have converged into the same values for the model parameters. The 95% Credibility Interval for the parameter is drawn as a thick black line. The numbers correspond to the endpoints of the interval. In this plot, a pre-treatment variable means a measurement from the RBCs generated before the patient started taking the CDK4/6is and a post-treatment variable means a measurement from the RBCs generated after starting the CDK4/6i. Whenever a pre-treatment and post-treatment variable are shown, they share the *x*-axis, and can be compared directly. The right columns show the Hamiltonian Monte Carlo chain mixing, which is a measure of convergence. No color is systematically above or below the others, which means that the probability space is being well-explored by the model. (**A**,**B**) Pre- and pos-treatment mean value of the mean corpuscular volume (MCV) across all patients. We highlight that the mean value of the MCV increased by 12.6 fL (95% CdI 13–14 fL) after the full RBC lifetime. (**C**,**D**) The pre- and post-treatment values of the red blood cell (RBC) count, which decreases with treatment. (**E**,**F**) Pre- and post-treatment value for mean corpuscular hemoglobin concentration. (**G**,**H**) Pre- and post-treatment hemoglobin concentration, which increases slightly with treatment. (**I**) The (pre-treatment) value of the estimated red blood cell lifespan in days. This model does not allow us to estimate the post-treatment RBC lifespan. Abbreviations: RBC—red blood cell, MCV—mean corpuscular volume, MCHC—mean corpuscular hemoglobin concentration, fL—femtoliter.

Bayesian credible intervals and their distributions for the most important population-level parameters are shown in Figure 2A–I and summarized in Table 1. Intervals and distributions between pre- and post-treatment parameters such as the hemoglobin value, the RBC count and the MCV are also shown.

The population mean for pre-treatment RBC counts was 87.8 fL (95% 86.7–88.9). After the pre-treatment RBCs were replaced, the mean MCV increased by 12.6 fL (95% CdI 13–14) to a value of 100.2 fL (95% CdI 99.0–101.4). The MCHC increased slightly by 0.69 g/dL (95% CdI 0.42–0.96) and the RBC count decreased by 0.77 × 10^9^/L (95% CdI 0.42 × 10^9^/L–0.96 × 10^9^/L). The net result was a 0.64g/dL (95% CdI 0.48–0.80) rise in hemoglobin. The mean total RBC lifetime was 118 (95% CdI 114–122) days, similar to the value measured experimentally in healthy subjects.

**Figure 3:**
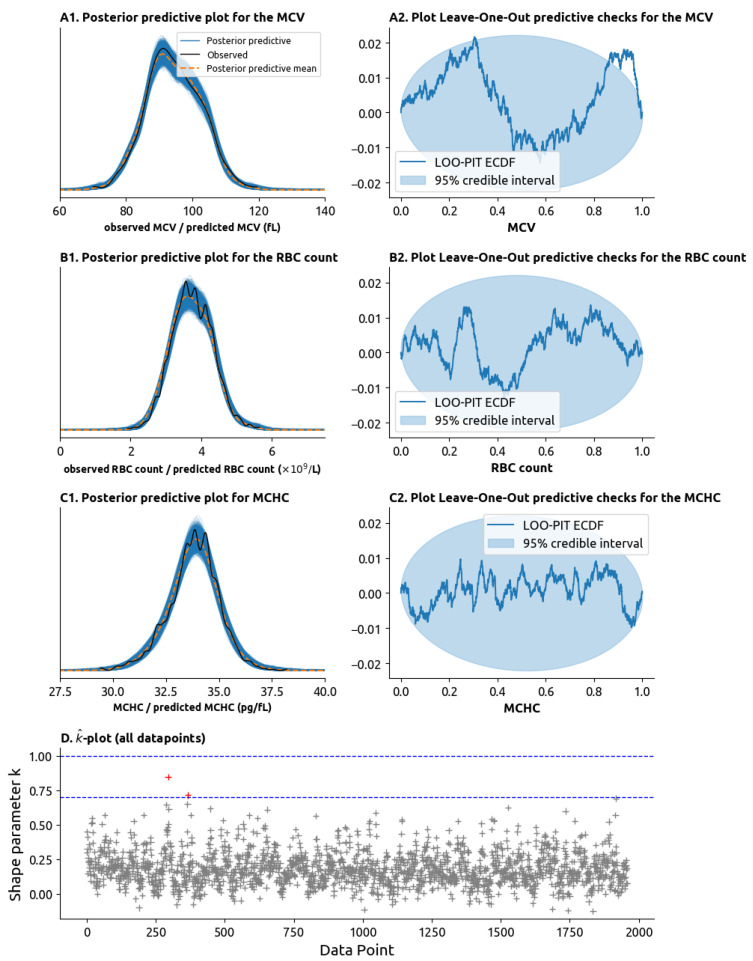
Model validity tests. (**A1**) Posterior predictive check (PPC) plot for the MCV. In a posterior predictive check plot, the MCV values randomly generated by the model are compared to the ones we observe in practice. The distributions of values generated by the model are drawn as multiple blue lines, and the mean of these values is drawn as the orange dotted dashed line. The distribution of the values observed in practice is drawn as the solid black line. If the black line remains close to the orange dotted line and inside the set of blue lines, then the model can be said to adequately explain the data. (**A2**) Leave-one-out (LOO) plot. The LOO plot tests for model validity by removing data points from the model and seeing how well the expected the datapoint given the remaining datapoints. Graphically, if the blue line is mostly inside the light-blue ellipsis, then the model passes the LOO check. (**B1**) PPC plot for the red blood cell (RBC) count. (**B2**) LOO plot for the RBC count. (**C1**) PPC plot for the mean corpuscular hemoglobin concentration (MCHC). (**C2**) LOO plot for the MCHC. (**D**) $\hat{k}$-plot (pronounced “k-hat” plot). Plots the $\hat{k}$ statistic on the y-axis for each of the 1959 laboratory measurements on the *x*-axis (each laboratory measurement combines the MCV, RBC count and MCHC). High values of the $\hat{k}$ statistic (above 0.7 or above 1.0, highlighted as dashed blue lines) represent outliers within an outsized impact on the model parameters which are not well explained by the data. The presence of only 2 values with $\hat{k}$ > 0.7 (in red) and no values with $\hat{k}$ > 1.0 mean that the datapoints are well explained by the model.

The model performed well in diagnostic checks. The posterior predictive checks (PPC) plots are contained (Figure 3(A1,B1,C1)) within the bounds defined for the 95% confidence bounds and the leave-one-out (LOO) plots (Figure 3(A2,B2,C2)) are mostly contained within the 95% confidence envelope. The $\hat{k}$ statistic for the 1959 data points (combining the RBCs, the MCV and the MCHC for each time point) showed that no outliers had a disproportionate impact on the model’s likelihood, with only two points with $\hat{k}>0.7$ and no data points with $\hat{k}$ > 1.0 (Figure 3D).

## 4. Discussion

Our analysis suggests that ribociclib, palbociclib, and abemaciclib do not decrease the RBC lifespan in pre-treatment erythrocytes for most patients in the doses currently used in clinical practice. The measured value for pre-existing RBC lifespan was 118 days, which is the same as the experimentally measured RBC lifespan by direct methods. This value has been determined to be approximately 118 days [15]. Unfortunately, our method cannot determine the lifespan of RBCs created during treatment. Although our laboratory data were collected from patients with metastatic breast cancer, there is no a priori reason to think that the results would be different for patients treated with CDK4/6is for breast cancer in the adjuvant setting or even for patients treated for other malignancies. Although we believe that this analysis should be repeated for patients being treated in the adjuvant setting, due to the more recent approval of CDK4/5is in this setting, we were unable to gather a large cohort of such patients.

The increase in MCV is counterbalanced by a decrease in RBC numbers, which results in a slight (and probably clinically insignificant) hemoglobin increase. It is not clear whether this slight increase in hemoglobin is due to the mechanism of action of the drugs, to better disease control, or whether it is within the expected intra-subject variability of hemoglobin concentration. The fact that the increase in the hemoglobin content of RBCs (due to a larger volume with a near-constant hemoglobin concentration) is almost exactly compensated by a decrease in RBC numbers to maintain a very similar hemoglobin concentration suggests that CDK4/6is do not interfere with the endogenous mechanisms for the regulation of total body hemoglobin concentration. The decrease in RBC numbers is not due to RBC destruction, because if it were, we would not expect hemoglobin to remain almost constant. The fact that hemoglobin remains almost constant (and even increases slightly) suggests that the decrease in RBC numbers is a result of the hematopoietic system attempting to compensate for the fact that new RBCs are larger and contain a higher amount of hemoglobin. This can be contrasted with what typically happens when RBCs are lost (from blood loss or hemolysis): when RBCs are lost, they are replaced with reticulocytes which do have a larger MCV but not a higher amount of hemoglobin. In our patients, we see RBCs being replaced with larger cells, but with a higher amount of hemoglobin. These findings suggest that these drugs also do not have a clinically significant adverse impact on RBC production or maturation, at least in clinically stable patients without baseline hematological disease or acute sickness.

Although we believe this work concludes to a reasonable degree of confidence that CDK4/6is do not cause clinically significant RBC fragility with a noticeable decrease in RBC lifespan, we cannot determine whether RBCs created under the effect of these drugs (that is, the new RBCs) are more or less fragile than the baseline RBCs (the old RBCs). To determine the RBC lifespan for the new RBCs, one would need a large population of patients who had stopped taking a CDK4/6i and who had not started any treatment with potential impact on RBC characteristics, such as RBC volume, and with no impact on the rate of RBC production. In breast cancer patients with metastatic disease, this situation is very unlikely, as many patients who experience disease progression under CDK4/6is will start cytotoxic chemotherapy, which has a range of hematological toxicities, making it almost impossible to interpret the rate of change of MCV as time passes due to an unpredictable impact in RBC characteristics and numbers. Few patients are treated with just endocrine therapy upon progression with a CDK4/6i [26].

However, with access to a large population of patients treated with CDK4/6is in the adjuvant setting, it would be possible to apply this approach. For example, further research could be conducted in the patient population of the active treatment arm of the NATALEE [7] or MonarchE [6] trials, in which breast cancer patients with resected localized breast cancer were treated with ribociclib together with hormone therapy for 3 years in the adjuvant setting. The patients who did not relapse during adjuvant treatment had no evidence of disease. Unlike patients in the metastatic setting, who most often start systemic treatment after stopping ribociclib, patients in the NATALEE or MonarchE trials who stopped the CDK4/6i were treated only with endocrine therapy, which has no relevant hematological toxicities. The time taken for the MCV of those patients to return to their baseline could be used to estimate the lifespan of the RBCs created during treatment with the CDK4/6i.

Despite these encouraging results, which suggest that CDK4/6is do not increase RBC fragility or decrease RBC lifespan, one should note that none of these patients had baseline acquired or congenital hemolytic anemia. This work does not prove that CDK4/6is are safe in patients with co-morbidities such as hemolytic anemia. Even if they do not increase membrane fragility, it is possible that they might hinder the bone marrow response to stress, which could cause a shift from compensated hemolysis to severe hemolytic anemia in a susceptible patient, which can happen in some inherited hemoglobinopathies [27].

We believe this work helps establish the safety of CDK4/6is in terms of adverse events that affect RBC production or RBC destruction. These findings may help guide experiments to better understand these hematological effects.

## 5. Conclusions

We were able to show, in a non-invasive way and using only widely available laboratory measurements, that the use of CDK4/6is in the doses commonly used to treat breast cancer is not associated with increased RBC destruction. The main limitation of our work is the fact that in our population, we could not assess the lifetime of the red blood cells created under the effect of CDK4/6is, but only the lifetime of RBCs created prior to the start of the CDK4/6is. Data regarding the lifetime of RBCs created under the effect of CDK4/6is could be gathered by studying patients using CDK4/6is as adjuvant treatment.

We believe this work helps establish the safety of CDK4/6is in terms of adverse events that affect RBC production or RBC destruction, and our findings may help guide experiments to better understand the mechanism behind these hematological effects and their clinical significance.

## Figures and Tables

**Table 1:** Clinico-demographic characteristics of patients. The top rows present the patient categories as an absolute number and as a percentage of the total (in parenthesis). The bottom rows show the numeric value as well as the interquartile range (in parenthesis). Age refers to the patient age at the start of treatment with the corresponding CDK4/6 inhibitor. To calculate continuous treatment duration, the treatment was assumed to be continuous if there was no treatment interruption of 30 days or more.

Category	N (%)
CDK4/6 inhibitor	122 (100%)
Abemaciclib	16 (13.1%)
Palbociclib	65 (53.3%)
Ribociclib	41 (33.6%)
Sex assigned at birth	
Female	122 (100%)
Male	0 (0%)
	**Median (inter-quartile range)**
Age	59 years (49–70)
Continuous treatment duration	7.2 months (3.4–13.3)

## Data Availability

The code and anonymized data are available upon request to the first author of the paper.

## Abbreviations

The following abbreviations are used in this manuscript:

CDK	Cyclin-dependent kinase
LOO	Leave-one-out (plot)
MCHC	Mean corpuscular hemoglobin concentration
MCV	Mean corpuscular volume
PPC	Posterior predictive check
RBC	Red blood cell
